# MicroRNA and tasiRNA diversity in mature pollen of *Arabidopsis thaliana*

**DOI:** 10.1186/1471-2164-10-643

**Published:** 2009-12-30

**Authors:** Robert Grant-Downton, Gael Le Trionnaire, Ralf Schmid, Josefina Rodriguez-Enriquez, Said Hafidh, Saher Mehdi, David Twell, Hugh Dickinson

**Affiliations:** 1 Department of Plant Sciences, South Parks Rd, Oxford OX1, 3RB, UK; 2 Department of Biology, University of Leicester, Leicester, LE1 7RH, UK; 3 Department of Biochemistry, University of Leicester, Leicester, LE1 7RH, UK; 4 Instituto Antonio González, Universidad de La Laguna, 38206 La Laguna, Santa Cruz de Tenerife, Spain

## Abstract

**Background:**

New generation sequencing technology has allowed investigation of the small RNA populations of flowering plants at great depth. However, little is known about small RNAs in their reproductive cells, especially in post-meiotic cells of the gametophyte generation. Pollen - the male gametophyte - is the specialised haploid structure that generates and delivers the sperm cells to the female gametes at fertilisation. Whether development and differentiation of the male gametophyte depends on the action of microRNAs and trans-acting siRNAs guiding changes in gene expression is largely unknown. Here we have used 454 sequencing to survey the various small RNA populations present in mature pollen of *Arabidopsis thaliana*.

**Results:**

In this study we detected the presence of 33 different microRNA families in mature pollen and validated the expression levels of 17 selected miRNAs by Q-RT-PCR. The majority of the selected miRNAs showed pollen-enriched expression compared with leaves. Furthermore, we report for the first time the presence of trans-acting siRNAs in pollen. In addition to describing new patterns of expression for known small RNAs in each of these classes, we identified 7 putative novel microRNAs. One of these, ath-MIR2939, targets a pollen-specific F-box transcript and we demonstrate cleavage of its target mRNA in mature pollen.

**Conclusions:**

Despite the apparent simplicity of the male gametophyte, comprising just two different cell types, pollen not only utilises many miRNAs and trans-acting siRNAs expressed in the somatic tissues but also expresses novel miRNAs.

## Background

In flowering plants, small RNAs below 30 nucleotides in size are essential and diverse components of the transcriptome [1]. Since their initial discovery in plants, small RNA systems have become better characterised and various classes of small RNA molecules with different functions are now recognisable. These functions include regulation of gene expression at transcriptional and post-transcriptional levels, repression of transposable element activity and defence against viral pathogens, and directed epigenetic changes in the genome such as DNA methylation and heterochromatin formation [1,2].

MicroRNAs are a distinct class of small RNAs derived from endogenous Pol II-transcribed genes. The transcripts from these genes form fold-back structures or hairpins that are recognised in the nucleus by the protein machinery of the microRNA pathway [1,2]. These hairpin transcripts are processed in specific bodies in the nucleus by proteins such as DICER-LIKE1 (DCL1) [3,4]. Here, a site-specific excision process removes a ~21 nucleotide duplex RNA from the stem of the hairpin structure. This RNA is then exported to the cytoplasm, where one strand binds the ARGONAUTE1 (AGO1) protein in the RISC complex. Subsequently, this protein-RNA complex interacts with mRNAs with high complementarity to the miRNA. In flowering plants, the Slicer endonuclease activity of the AGO1 protein acts to cleave the mRNA where it binds the miRNA [5]. Subsequently, the cleaved mRNA is routed into degradation pathways. In this manner, not only is the translation of the mRNA prevented but the levels of the mRNA target are down-regulated. In plants, it has now been shown that microRNAs also act to repress translation without cleavage of the mRNA, a regulatory mechanism that appears to involve not only AGO1 but also AGO10 and probably another member of the ARGONAUTE family [6]. Over 100 microRNAs have now been described in *Arabidopsis thaliana *and these are known to directly regulate a large number of transcripts, in particular those that encode key regulatory proteins such as transcription factors and F-box proteins that regulate entry of other proteins into the ubiquitin-dependent degradation pathway.

Certain miRNAs do not target coding mRNAs but instead recognise long non-coding transcripts from so-called *TAS *loci [2]. These miRNAs are homologous to one or more typically two different sites on the target *TAS *transcript. These sites act as recognition points from which further small RNAs are formed [7]. After cleavage to generate two *TAS *fragments, the *TAS *RNA is further processed to generate secondary small RNAs [8]. An RNA-dependent RNA polymerase enzyme, RDR6, is then recruited to synthesise a complementary strand and the resulting duplex RNA is sequentially cut by the DICER-LIKE 4 DCL4 enzyme to form phased 21 nt small RNAs called trans-acting siRNAs [9,10]. The exact mechanism by which a single conventional cleavage event directed by a microRNA recruits RDR6 and DCL4 activity to generate secondary siRNAs from *TAS *RNAs remains poorly understood [11]. Secondary siRNAs are known to be only rarely produced after conventional miRNA cleavage events on coding transcripts [12].

In plants, further classes of small RNAs are produced from RNAs derived from non-coding regions of the genome by various mechanisms, from both PolII and PolII-independent sources [13,14]. These small RNAs have a major role in establishing and maintaining patterns of DNA methylation and heterochromatin architecture throughout the genome [13,14].

Recent innovations in sequencing technology [15] have allowed extensive surveys of small RNA populations in flowering plants, notably in the model species *Arabidopsis thaliana*. However, until recently much of this work was focused on collectively sequencing the small RNAs from complex aggregates of sporophytic tissues to whole plants from both wild type and mutant lines. Notably absent has been a focus on the reproductive cells and the highly reduced post-meiotic male and female gametophytes (pollen and embryo-sacs). In flowering plants, gametes are not directly formed from the meiotic products but instead arise from a limited number of haploid mitotic divisions. In the male gametophyte, the four products of meiosis each undergo two further mitotic divisions. The first division is highly asymmetric and forms a larger vegetative cell and a smaller generative or germ cell. The germ cell undergoes a further division to form a pair of sperm cells that are suspended within the vegetative cell cytoplasm. In Arabidopsis, mature pollen grains at the point of release from the anthers consist of three cells of these two distinct cell types.

Previous work has questioned whether small RNA pathways are maintained and function as the male gametophyte develops [16]. However, it is clear from recent investigations that small RNA pathways are not down regulated, but transcript levels of various small RNA pathway genes show dynamic and complex changes during male gametophyte development [17]. It is evident that cell-specific differences contribute to these dynamic changes as sperm cells are significantly enriched for some transcripts, such as *AGO5*, compared to whole mature pollen [18]. Recent work has shown that silencing of repetitive DNA and transposons is weakened in the vegetative cell leading to the formation of 21 nt small RNAs from their transcripts that are claimed to reinforce silencing in the associated sperm cells [19]. A functioning microRNA pathway in mature pollen has been confirmed despite suggestions that mature microRNAs may not be produced from their precursors in late pollen development [16]. Not only have several families of microRNAs with a known function in the somatic phase of development been identified in pollen by *in situ *hybridisation [20,21], but many more have been discovered by RT-PCR analysis and the use of a modified 5' RACE protocol that has demonstrated the precise cleavage of transcripts targeted by several different microRNAs [17]. Thus, microRNAs and other small RNAs are likely to make a major but hitherto unappreciated contribution to pollen gene expression patterns.

We have utilised 454 sequencing to explore the diversity of small RNAs in mature pollen of *A. thaliana*. We describe the general composition of the small RNA population present in mature pollen and focus on the diversity of microRNAs and tasiRNAs, as these small RNAs have a direct effect on the abundance and translation of target mRNAs. We identify known microRNAs and tasiRNAs and also survey the sequence information for putative novel microRNAs. We independently verify the expression in pollen and relative abundance of selected microRNA sequences identified by 454 sequencing using quantitative RT-PCR. Amongst the candidate novel microRNAs, we confirm that one, named ath-MIR2939, cleaves its predicted target transcript, At3g19890. This corresponds to a pollen-specific F-box family transcript that is significantly enriched in sperm cells. Its discovery supports the assertion that novel miRNAs exist with putative roles in regulating gametophyte-specific transcripts that may be of key importance in reproduction.

## Results

### The pollen 454 sequencing dataset

Among the 32139 unique sequences between 15 and 30 nt, the majority are between 19 and 24 nt in length - the usual range size of small RNAs in plants. In terms of sequencing depth, more than 86.5% of these sequences are represented by only 1 read, 12.5% by 2 to 10 reads while the remaining 1% are represented by more than 10 reads. This indicates that our data are mainly qualitative and demand further verification and quantification, at least for specific microRNAs. BLAST analysis on Arabidopsis chromosomes indicates that 41% of the 32139 unique sequences perfectly or nearly perfectly (one mismatch) match the genome, which corresponds to 55% of the total reads (Tables 1 &2). However, around 60% of the unique sequences show 2 mismatches or more indicating that the rate of sequencing mistakes is probably relatively high. The short lengths of the sequences thus make the analysis difficult because mismatching at this level can generate several ambiguous potential hits. To avoid mis-identification of some small RNAs we decided to use only the 13048 sequences showing 0 or only 1 mismatch to the genome for further BLAST analysis.

**Table 1:** 454 sequencing results

	Unique sequences	Proportion	Reads
**Sequences between 15 and 30 nt**	32129	100%	54764
			
**Sequences with a Blast result**	31125	96.9%	53366
**Perfect match**	11249	35%	26290
**One mismatch**	1799	5.6%	3309
**Two mismatches or more**	18077	56.3%	23767

Sequences between 15 and 30 nt were used for Blastn analysis of the *Arabidopsis *genome sequence. The numbers of unique sequences scoring a Blast hit (with 0, 1, or more than 1 mismatch) are indicated, together with read numbers.

**Table 2:** Number of small RNA sequences between 15 and 30 nt length

Size class	Number of reads
**15**	3883
**16**	7657
**17**	4664
**18**	5156
**19**	4898
**20**	4179
**21**	3648
**22**	3725
**23**	3468
**24**	3582
**25**	3239
**26**	2606
**27**	1914
**28**	1157
**29**	635
**30**	353

Total number of sequences for each size class between 15 and 30 nt in length.

### Global BLAST analysis

BLAST analysis of the 13048 sequences was performed in both genic and intergenic nucleotide databases (Table 3). 931 sequences recorded hits in the two distinct databases with exactly the same score and it was thus impossible to assign them to either a genic or an intergenic location. 42.6% of the sequences were located to intergenic sequences, which makes them candidates for siRNAs associated with silencing of these regions through DNA methylation and chromatin modification. Among 50.3% of sequences matching the genic part of the genome, around 1% are associated with post-transcriptional silencing RNAs (miRNAs and tasiRNAs), the focus of this paper. Close to 19.5% (2537) of identified small RNAs correspond to protein-coding genes suggesting the regulation of the corresponding transcripts by silencing RNA pathways (see below). Other sequences exactly match transposable element (TE) genes, which agrees with reports of abundant siRNAs silencing TEs in Arabidopsis mature pollen [19]. A significant proportion (26.4%) of the small RNA sequences share homology with genic parts of the genome corresponding to ubiquitous and highly expressed structural RNAs such as rRNA or tRNA. This is a common occurrence in small RNA sequencing analyses, presumably arising from their abundance as normal cellular degradation products. A very small proportion of small RNAs (0.8%) correspond to mitochondrial small RNAs and less than 0.3% correspond to chloroplastic small RNAs.

**Table 3:** General Blastn results

	Perfect match		1 mismatch		Total		
	Nb	Reads	Nb	Reads	Nb	%	Reads
**Silencing RNAs**							
*miRNA*	71	209	14	22	85	**0.7**	231
*TAS genes*	41	65	8	8	49	**0.4**	73
**Candidate silencing RNAs**							
*Protein-coding genes*	2114	2487	423	1025	2537	**19.4**	3512
*Transposon*	172	178	51	71	223	**1.7**	249
*Pseudogenes*	15	33	5	5	20	**0.2**	38
**Non-protein coding RNAs**							
*rRNA*	2305	4396	232	258	2537	**19.4**	4654
*tRNA*	737	7568	173	811	910	**7**	8379
*snoRNA*	20	21	1	1	21	**0.2**	22
*snRNA*	6	7	1	1	7	**0**	8
*Other RNAs*	26	56	7	8	33	**0.2**	64
**Organellar small RNAs**							
*Mitochondrial*	58	84	42	47	100	**0.8**	131
*Chloroplastic*	16	26	19	26	35	**0.3**	52
**Intergenic regions**	4933	9540	627	746	5560	**42.6**	10286
**Undetermined**	735	1620	196	280	931	**7.1**	1900

Sequences with 0 or 1 mismatch with the *Arabidopsis *genome were compared with the intergenic and "genic" databases. Some matched silencing RNAs (miRNAs and TAS genes), candidate silencing RNAs (protein-coding genes or transposons), non-protein coding RNAs (mainly rRNAs and tRNAs) and organellar small RNAs. Other sequences either matched intergenic regions, or could not be assigned to either region. Nb: number of unique sequences

### Identification and quantitative RT-PCR validation of known microRNAs

BLASTN analysis of the 31239 unique sequences with 0, 1 or 2 mismatches led to the identification of 32 known Arabidopsis microRNAs corresponding to 396 reads (Table 4). Some miR* instead of, or in addition to the miR were also identified for the miR827, miR396 and miR829 families. The major proportion of the identified microRNAs target transcription factors, with 63 reads (16%) for miR156/157 (targeting *SPL *family), 38 reads (9.6%) for miR172 (targeting *AP2 *family) and 26 reads (6.6%) for miR159 (targeting *MYB/TCP *family). Another important category of identified microRNAs targets the PPR protein family with 65 reads (16.4%) for miR158 and 45 (11.4%) for miR161.

**Table 4:** Known *Arabidopsis *microRNAs identified in Col-0 mature pollen

	Perfect match		1 or 2 mismatches		
	Exact	Shorter	1	2	Total
**miR156**	5	26	21	7	*59*
**miR157**	1	2	0	1	*4*
**miR158**	42	1	15	7	*65*
**miR159**	18	3	3	2	*26*
**miR160**	2	0	1	1	*4*
**miR161**	18	5	17	5	*45*
**miR162**	0	0	1	0	*1*
**miR164**	0	1	0	*1*	2
**miR165**	0	1	0	1	*2*
**miR166**	0	0	0	1	*1*
**miR167**	0	1	0	0	*1*
**miR168**	15	0	4	0	*19*
**miR171**	0	2	0	0	*2*
**miR172**	2	20	13	3	*38*
**miR173**	0	9	2	2	*13*
**miR319**	1	0	0	1	*2*
**miR390**	0	3	0	0	*3*
miR396/miR396*	1	6	2	0	*9*
**miR399**	3	2	0	3	*8*
**miR400**	0	1	1	1	*3*
**miR403**	1	1	3	1	*6*
**miR472**	0	1	0	0	*1*
**miR776**	0	0	1	0	*1*
**miR778**	0	3	1	0	*4*
**miR780**	0	0	3	0	*3*
**miR824**	1	0	2	0	*3*
miR827*	0	1	0	0	*1*
miR829/miR829*	2	9	1	1	*13*
**miR845**	6	3	2	0	*11*
**miR859**	16	0	16	13	*45*
**miR869**	0	0	0	1	*1*
**Total**	**134**	**101**	**110**	**51**	**396**

A separate Blastn analysis of all sequences against the *Arabidopsis *microRNAs database has been performed to add to the list of identified miRNAs first generated by the global Blastn analysis. Shorter sequences as well as sequences showing 2 mismatches have been included. This resulted in the detection of 32 different miRNAs or miRNAs* in Col-0 mature pollen.

Interestingly, several components of the different epigenetic pathways seem to be targeted by microRNAs in pollen. miR168 (19 reads) and miR403 (7 reads) were detected, targeting *ARGONAUTE1 *and *ARGONAUTE2 *respectively. One single read of miR162 which targets *DCL1 *has also been detected. MiR773 and miR778 that target methyltransferases (*MET2*, a DNA methyltransferase, and two *SUVH *histone methyltransferases respectively) are also present. The remaining microRNAs target genes involved in hormone response like *AUXIN-RESPONSE FACTORs *(miR160 and miR167, 5 reads), genes involved in general metabolism or those having no identified targets.

miR173 and miR390 (16 reads) that target *TAS *genes are also detectable which suggests activity of the tasiRNA pathway, where initial cleavage of the *TAS *transcripts by these miRNAs is required to initiate tasiRNA biogenesis. Nine of the 33 detected microRNAs were singletons, which leaves open the possibility that their expression levels are either similar, or that the number of sequencing reads in this study was insufficient to resolve differences in their expression levels. Digital gene expression of small RNAs has recently been demonstrated to generate systematic and reproducible biases [22], highlighting the importance of further quantitative analysis by RT-PCR to confirm relative abundance of small RNAs.

**Figure 1:**
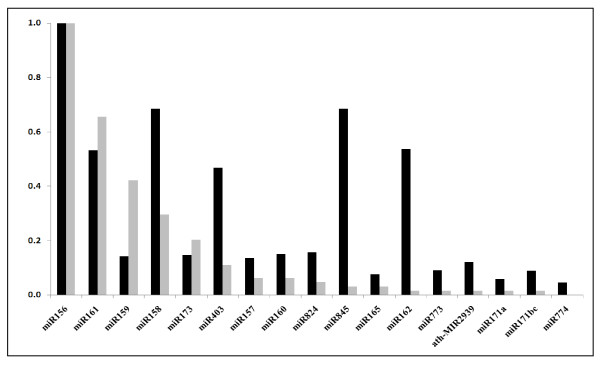
**Comparative quantification of microRNA abundance in *Arabidopsis *Col-0 mature pollen using 454 sequencing and q-RT-PCR**. MicroRNA expression levels (as determined by 454 sequencing and Q-RT-PCR) were normalized to that of miR156 - the most abundant microRNA. Black columns, Q-RT-PCR values; grey columns, 454 sequencing results.

**Figure 2:**
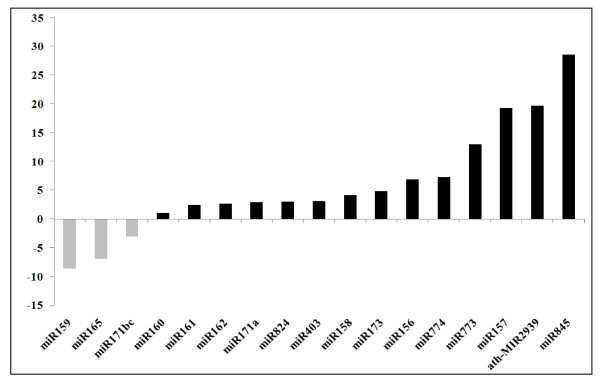
**Relative abundance of miRNAs between gametophytic and sporophytic tissues**. MicroRNA expression in Col-0 leaf and pollen determined using Q-RT-PCR. Y-axis, fold-change in expression between gametophyte and sporophyte. Grey columns, leaf enriched microRNAs; black columns pollen-enriched microRNAs.

We performed qRT-PCR on a subset of 17 microRNAs, ranging from those frequently detected (miR156 and miR161, with respectively 59 and 45 reads) to singletons (miR162, miR171a, miR171bc, and miR773), and including those showing 1 or 2 sequence mismatches (miR162, miR165, miR173 and miR773, see Additional file 1: Table S1). Since all of these microRNAs amplified (see Additional file 1: Figure S1) at high PCR efficiency values, it follows that 454 sequencing generates significant sequencing mistakes. For this reason we consider that sequences showing 1 or 2 mismatches (when compared with the *Arabidopsis *genome) are likely to represent their corresponding authentic miRNAs. We also detected miR774 by RT-PCR, even though this sequence was absent from our own 454, and indeed all available online datasets http://asrp.cgrb.oregonstate.edu/db/. These data suggest that our sequencing depth provided insufficient coverage to identify all pollen small RNAs. Nevertheless, quantification revealed that the most abundant miRNA in our 454 dataset, miR156, was also detectable by Q-RT-PCR. The relative abundance of all other miRNAs was normalized to miR156 for both miRNA Q-RT-PCR and sequencing data (Figure 1). Overall there are few significant differences between quantitative RT-PCR and sequencing, and the major differences concern the relative abundance of miR159, which is over-estimated, and miR403, miR845 and miR162 which are under-estimated by sequencing. The relative abundance of the remaining microRNAs appears in a similar rank order in both assays (Figure 1). This additional step is thus important to verify microRNA expression levels independently within samples. We also compared miRNA expression between sporophytic and gametophytic samples by quantifying miRNA abundance in Col-0 leaf RNA. Our data indicate that 3 microRNAs, miR159, miR165, miR171b,c are enriched in the sporophyte while the remaining 14 microRNAs were pollen enriched. miR157, miR845 and ath-MIR2939 were not amplified from leaf material and may therefore be pollen-specific, or very strongly pollen enriched (Figure 2).

### Putative novel microRNAs

**Figure 3:**
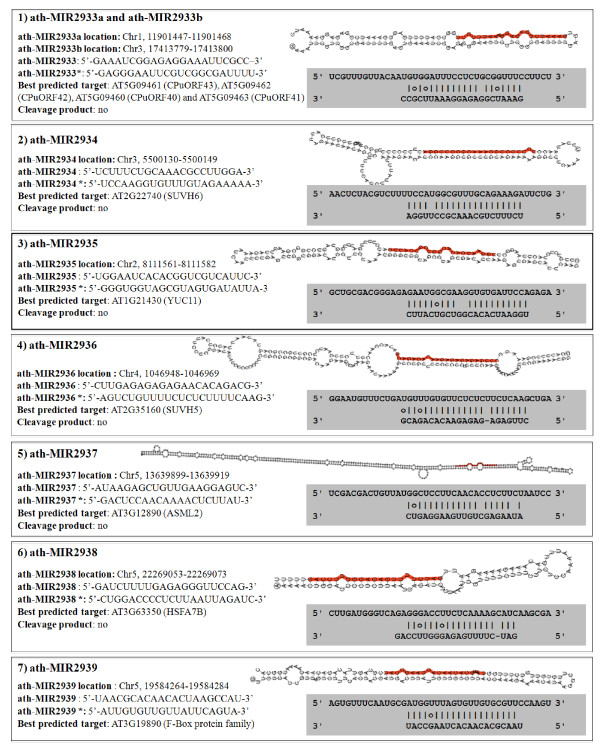
**Putative new miRNAs detected in Col-0 mature pollen**. 7 putative candidates with hairpin structure and a predicted mRNA target identified by MiRCaT software. The sequence of the putative corresponding miRNA* is also shown.

The use of the MiRCat and Target prediction software permitted us to detect putative new microRNA candidates (Table 5). Seven of these ten putative microRNAs fufil the criteria required for designation as a valid microRNA, as set out in [23] since a convincing hairpin structure is predicted for these candidates (Figure 3). One candidate, ath-MIR2933, seems to have 2 different genomic locations and could target CPUORFs and a transcription factor. Derivation of the same mature microRNA sequence from two or more independent genomic loci is common in plant microRNAs, for example miR160. Other candidates have only one genomic location and are predicted to target transcription factors (ath-MIR2937, ath-MIR2938 and ath-MIR2939), and *SUVH6*, a histone methyltransferase (ath-MIR2934). Those remaining putatively target a translation initiation factor (ath-MIR2936) and *MEI1 *and a monooxygenase (*YUC11*) (ath-MIR2935).

**Table 5:** Putative new microRNAs

sRNA	Sequence	Target	Accession	Cleavage
ath-MIR2933a	GAAATCGGAGAGGAAATTCGCC	CPUORF	AT5G09460	Not tested
ath-MIR2933b	GAAATCGGAGAGGAAATTCGCC	CPUORF	AT5G09460	Not tested
ath-MIR2934	TCTTTCTGCAAACGCCTTGGA	SUVH6	AT2G22740	No
ath-MIR2935	TGGAATCACACGGTCGTCATTC	YUC11	AT1G21430	No
ath-MIR2936	CTTGAGAGAGAGAACACAGACG	TIF	AT1G76720	No
ath-MIR2937	ATAAGAGCTGTTGAAGGAGTC	ASML2	AT3G12890	No
ath-MIR2938	GATCTTTTGAGAGGGTTCCAG	HSFA7B	AT3G63350	No
ath-MIR2939	TAACGCACAACACTAAGCCAT	F-Box	AT3G19890	Yes

7 candidate sequences capable of forming hairpin structures identified by miRCat software. Target sequences are shown, and cleavage was tested for 5 of the 7 sequences using 5' RACE-PCR.

### Detection of miRNA-derived cleavage products by modified 5' RACE-PCR

To validate whether these putative microRNAs actually cleave the computationally predicted target mRNAs *in vivo*, several of the predicted target mRNAs were tested using a modified 5' RACE procedure that allows detection of uncapped transcripts produced after miRNA-directed cleavage. To validate cleavage, we used gene-specific nested primers located a short distance 3' upstream of the predicted site of small RNA/mRNA binding. Of the putative target mRNAs tested, only one (At3g19890, an F-box family transcript) produced a cleavage product that corresponded to the predicted cleavage product from ath-MIR2939 activity (Figure 3). This result clearly validates ath-MIR2939 as a novel microRNA and the expression of At3g19890 appears to be restricted to the male gametophyte according to microarray data (see Additional file 1: Table S2). To test independently whether this microRNA could have other targets, the mature microRNA sequence was used to query an online database of computationally predicted microRNAs and their targets ([24], http://sundarlab.ucdavis.edu/mirna/). This database predicted the existence of this novel microRNA and its targeting of At3g19890, but also predicted targeting of another F-box transcript, At3g17265. However, we were unable to confirm cleavage of At3g17265 in mature pollen.

**Figure 4:**
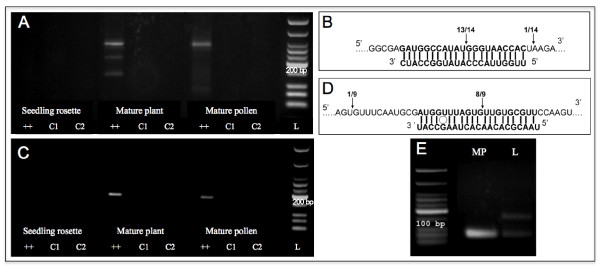
**MicroRNA induced cleavage of At3g19890; detection of cleavage products by modified 5' RACE**. **A**. 5' RACE to identify miR774a-directed cleavage products. **B**. Watson-Crick base pairing and pattern of cleavage of At3g19890 by miR774a. **C**. Gel image showing products of 5' RACE reaction to detect ath-MIR2939-directed cleavage. **D**. Watson-Crick base pairing and pattern of cleavage of At3g19890 by ath-MIR2939. **E**. RT-PCR detection of mature ath-MIR2939 in mature pollen (MP) and leaf (L). For A and C, Lanes C1 and C2 are controls (minus 5' RACE adapter primer and minus gene specific primer). L, DNA ladder.

Some of the putative microRNAs shared predicted mRNA targets with known, microRNAs also identified in our pollen small RNA sequencing dataset. For example ath-MIR2934 is predicted to target *SUVH6*, which is also targeted by miR778 identified by sequencing. At3g19890 was a confirmed target of ath-MIR2939 but is also predicted to be a target of miR774. Interestingly miR774 was identified as pollen-enriched in Q-RT-PCR analysis (Figure 2). 5' RACE revealed that *SUVH6 *transcripts appeared to be cleaved within the miR778 binding site identified, although other uncapped transcripts were also identified (see Additional file 1: Figure S2). Nevertheless, cleavage product was not detected at the predicted site for ath-MIR2934. The transcriptional profiles from microarray analysis for *SUVH6 *during pollen development showed that transcript levels peak in tricellular pollen and remain at similar levels in mature pollen (see Additional file 1: Table S2). 5' RACE analysis also confirmed that miR774 was present in pollen as the predicted cleavage product of At3g19890 was detected, exclusively in pollen and flowering plant material (Figure 4).

In mature pollen, cleavage products for *SUVH5 *were not detected in the predicted binding site of ath-MIR2936. In contrast to *SUVH6 *they were not found for the miR778 site either.

### Identification of tasiRNAs in pollen

The analysis of our pollen small RNA sequence dataset with pssRNAminer identified clusters of small RNAs that exactly matched some transcripts, but in a phased manner, which clearly revealed the presence of trans-acting siRNAs (Table 6). Our analysis excluded all clusters corresponding to non-nuclear small RNAs, structural RNAs and protein-coding transcripts to allow the identification of true tasiRNAs (phased siRNAs derived from long non-coding RNAs). Phased small RNAs from four different previously described *TAS *genes (*TAS1a, b *and *c *and *TAS2*) are represented, having around 4 to 10 small RNAs matching their sequence, in both strands (see Additional file 1: Figure S3). Both *TAS1 *and *TAS2 *transcripts are first cleaved by miR173 to initiate the generation of phased siRNAs. The presence of this microRNA in our dataset thus confirms that conventional tasiRNA pathways are functional in mature pollen. However, while we were able to detect tasiRNAs in mature pollen by RT-PCR, their precursor TAS transcripts proved no longer detectable - although these were readily amplified from a control vegetative sample (see Additional file 1: Figure S4).

**Table 6:** **Mature pollen trans-acting siRNAs**. 4 transcripts with features of trans-acting siRNAs identified by pssRNAminer software. The number of phased and non-phased small RNAs (both strands; +1 and -1) are indicated.

		Phased small RNAs		Non-phased small RNAs	
AGI number	Gene Name	+1	-1	+1	-1
AT2G27400	TAS1A	5	5	2	0
AT1G50055	TAS1B	7	1	0	0
AT2G39675	TAS1C	3	4	2	2
AT2G39681	TAS2	2	2	1	2

### Conservation of microRNA expression in tobacco pollen

To examine whether miRNA expression in pollen is conserved in a species with bicellular pollen at dehiscence, a sample of highly conserved miRNAs were amplified from *Nicotiana tabacum *together with a control small RNA (NSRII) (see Additional file 1: Figure S5). All miRNAs detectable in *A. thaliana *tricellular pollen could also be detected in *N. tabacum *mature pollen - with the exception of miR159.

## Discussion

The use of new generation sequencing technologies to survey small RNA populations in flowering plants has largely focused on whole plants or aggregates of sporophytic tissues. A major omission has been the specific study of small RNA populations in reproductive tissues and cells. For this reason we have isolated mature pollen from *Arabidopsis thaliana *and utilised 454 technology to sequence the small RNA component of the transcriptome. The existence of a active late pollen small RNA transcriptome had remained in doubt following an early analysis of small RNA pathway gene expression [16], but subsequent studies have detected a range of small RNAs in late pollen development [20,21,17,19].

### Known microRNAs in pollen

We show here that microRNAs are a diverse component of the late pollen small RNA transcriptome. Previous work has shown that several known families of microRNAs could be detected in mature pollen by a modified RT-PCR amplification system [17] but by direct sequencing of small RNAs we have demonstrated 33 families of previously described microRNA families to be present in mature pollen. All of those found by the previous RT-PCR analysis were confirmed by this sequencing approach; indeed, the sensitivity of the 454 sequencing approach is such that one miRNA, miR319, undetectable in the RT-PCR analysis, was identified. This discovery of mature miR319 in pollen confirms a recent analysis of miR319a promoter expression patterns [25].

Given that microRNAs have only recently been discovered in pollen, there is as yet no direct evidence for a specific role for any microRNA in pollen development. However, it has been proposed [17] that microRNA function is necessary for pollen development given the presence and functioning of miR168 which operates to modulate *ARGONAUTE1 *transcript levels in a mechanism maintaining AGO1 protein levels by a feedback loop [2,26]. AGO1 appears to be necessary to make normal functional male gametophytes [27]. Mir160 may also be an important contributor to transcriptomic changes in pollen development, playing a part in auxin-regulated development through the elimination of *ARF16 *and *ARF17 *transcripts after pollen mitosis II.

Our results emphasise the potential importance of miRNAs in pollen development and maturation. For example transcriptomic analysis has shown that many transcription factors exhibit dynamic gene expression changes during male gametophyte development [28,29] and miRNAs that target transcription factors are well represented in our sequences (over one third of the families identified). Given the presence of miRs targeting transcription factor families such as *SPL *(miR156/miR157), *MYB/TCP *(miR159, miR319), *ARF *(miR160, miR167), *AP2 *(miR172), and *GRF *(miR396) there can be no doubt that miRs modulate the expression of many transcription factors during later stages of pollen development.

The other major classes of genes regulated by microRNAs are those involved in protein degradation (the F-box family and E2-ubiquitin conjugating enzyme family) and pentatricopeptide repeat (PPR) family. F-box family proteins, a large super- family in plants with 694 genes identified in the *Arabidopsis thaliana *genome alone [30], have a major role in targeting proteins for destruction via the ubiquitination pathway [31]. Indeed, F-box proteins are strikingly enriched in the sperm cell transcriptome [18] and recently an F-box protein (FBL17) was found to be necessary for pollen mitosis II and sperm cell formation [32]. If there is a need to remove specific F-box transcripts rapidly at this stage of development, it is perhaps not surprising that a microRNA that targets F-box family transcripts, miR859, is one of the most abundant in the mature pollen small RNA transcriptome; however, *FBL17 *itself does not appear to be targeted by a microRNA. The presence of miR399, which targets transcripts of the E2-ubiquitin conjugating enzyme family, also supports the idea that small RNA regulation of the protein degradation pathways is important in late pollen. The PPR proteins are another large family with significant roles in plastid and mitochondrial development in plants (reviewed in [33], and [34]). They are also known to play an important part in regulating male fertility (e.g. [35]). Their biochemical roles in these organelles include regulating of RNA stability (e.g. [36]) and RNA splicing (e.g. [37]). The relative abundance of microRNAs targeting *PPR *transcripts at this stage of development is interesting, as drastic changes in mitochondrial and plastid development, numbers and function occurs in the male gametophyte. In most flowering plants, these organelles are inherited uniparentally through the female and the male organelles are eliminated by different mechanisms (reviewed by [38]); it is plausible that dynamic changes in PPR transcripts and protein levels generated by microRNAs may contribute to this process.

MicroRNAs that target different epigenetic pathway transcripts are also represented in the mature pollen small RNA transcriptome. Our results confirm that the homeostatic feedback loop that regulates steady-state levels of AGO1 and DCL1 proteins is likely to operate, as both miR168 and miR162 were found. Interestingly, miR402 that regulates *ARGONAUTE2 *(*AGO2*) was also present, although RT-PCRs were not able to detect *AGO2 *transcripts during male gametophyte development [17], nor was *AGO2 *detectable in sperm cells [18]. This apparent anomaly may be explicable as recent computational work indicates that miR402 may target other mRNAs [39]. Another notable discovery was the relative abundance of miR778 in pollen. miR778 targets two histone methyltransferase transcripts *SUVH5 *and *SUVH6*. These proteins have roles in regulating histone methylation and silencing repeats and transposons [40,41]. Given reports [19] that the epigenetic silencing of such sequences is derepressed in the vegetative cell of mature pollen (primarily attributed to down-regulation of *DDM1*), the possibility of microRNA-directed down-regulation of these two transcripts is intriguing. Indeed, *SUVH5 *transcripts decrease from relatively high to undetectable levels after pollen mitosis II during male gametophyte development (see Additional file 1: Table S2), and differential levels between sperm cell and vegetative cell can be detected for *SUVH5*, with enrichment in sperm compared to mature pollen [18]. It is possible that miR778 may be acting to suppress these transcripts, perhaps in the vegetative cell alone - as seen with *DDM1*.

Remarkably, we did not find the highly conserved miRNAs miR855 and mir854 that are derived from retrotransposons at multiple genomic sites [42]. Given that miR854 and miR855 are derived from retrotransposons reported to be transcriptionally reactivated in the vegetative cell [19], their absence from our dataset is perplexing. Possibly these retrotransposon transcripts are routed into another small RNA pathway and consequently are not processed by the microRNA pathway.

The model plant *Arabidopsis thaliana *has tricellular pollen, with pollen mitosis II and sperm cell formation prior to pollen release from the anthers. Some species produce bicellular pollen, where pollen mitosis II and sperm cell production only occur after pollen germination in the pollen tube. Here, we used the mature pollen of tobacco to determine the expression of several conserved microRNAs in another dicotyledonous flowering plant with bicellular pollen at pollen release. Of the conserved microRNAs tested that were known to be present in *A. thaliana *pollen, only miR159 could not be amplified - although miR159 transcripts were amplified in a flower bud control. It is possible that miR159 is not expressed until after pollen mitosis II in the bicellular pollen of tobacco, when its targets such as *DUO1 *have completed their developmental function [43].

### Known trans-acting siRNAs

In the analysis of microRNAs, we found that miR173 was present in mature pollen. As four non-coding loci, *TAS1a*, *TAS1b*, *TAS1c *and *TAS2*, are the targets of miR173 and templates for the production of trans-acting siRNAs, we investigated whether tasiRNAs could also be detected in the mature pollen. Using pssRNAMiner, we found that phased siRNAs from all four *TAS *loci targeted by miR173 could be detected. This is clear evidence that not only is the miRNA pathway functional in pollen, but the tasiRNA pathway is also operating - a view supported by previously-reported evidence for *DCL4*, *RDR6 *and *SGS3 *expression during male gametophyte development [17].

In pollen, the roles of tasiRNAs remain obscure. Even their function in the sporophyte appears to be subtle as mutants such as *rdr6 *that eliminate detectable tasiRNA levels do not display dramatic phenotypes (e.g. [9]). Targets for *TAS1 *siRNAs are *PPR *and unclassified transcripts, whilst *TAS2 *tasiRNAs target *PPR *transcripts alone - again suggesting that tight regulation of *PPR *transcripts is important for normal pollen development, with considerable redundancy between miRNA and tasiRNA regulation of *PPR *transcripts.

### Novel microRNAs and tasiRNAs

The poor sampling of male gametophyte small RNAs to date and the unique gene expression profile of this developmental stage suggested that our dataset was likely to contain novel small RNAs. Further, a recent screen of Ds insertional mutants in *A. thaliana *for defects in male gametophyte development and function [44] resulted in 7 lines being mapped to intergenic or unannotated regions of the genome, hinting that some of these loci may generate small RNAs with specific roles in the male gametophyte. We therefore screened our dataset for potential microRNAs using the online UEA plant sRNA toolkit http://srna-tools.cmp.uea.ac.uk/cgi-bin/input_form.cgi?tool=mircat and a database of computationally predicted miRNAs [24]. Criteria required of a 'true' microRNA have recently been described [23], and application of these criteria resulted in our identification of several novel microRNA candidates.

To explore whether any of these candidate microRNAs were able to cleave their predicted target transcripts, modified 5' RACE experiments were performed on selected transcripts. Only one such microRNA, ath-MIR2939, was found capable of directing cleavage of its predicted target, an F-box family transcript At3g19890. Predicted to be functional [24], ath-MIR2939 is likely to be restricted to pollen as it has not been identified in other reported sequencing exercises, and it is not significantly amplified in leaf tissue by Q-RT-PCR. Furthermore At3g19890, the transcript ath-MIR2939 was most confidently predicted to target, appears to be restricted to male gametophyte development (see Additional file 1: Table S2), peaking in expression at the microspore stage. It is also enriched in sperm cells in mature pollen [18] although undetectable in whole pollen by RT-PCR. We show here shown that ath-MIR2939 induces cleavage of At3g19890 and must be involved in regulating this transcript *in vivo *at the post-transcriptional level. Additionally, this transcript is also targeted by miR774 which we also detected in mature pollen by Q-RT-PCR, suggesting that post-transcriptional regulation of this transcript may be functionally important during pollen development or fertilisation.

Our results also reveal the presence of known miRNAs targeting pollen-held transcripts, but without cleavage products being generated. However, this absence of cleavage is understandable in the context of recent work which shows that plant microRNAs can induce translational suppression [6,45]. In mature pollen, translational suppression may thus operate to inhibit production of protein products from stored transcripts (or transcripts in the process of storage), enabling transcripts to be accumulated during pollen maturation without concomitant protein synthesis. Protein production could subsequently be initiated only in the correct developmental context - such as water availability or perception of signals from stigmatic tissue. It is also possible that some late pollen microRNAs do not act gametophytically but are transmitted in fertilisation and subsequently regulate transcripts in the zygote and endosperm. An important precedent is the pollen *SSP *transcript in *Arabidopsis*, which is untranslated in the pollen but on transmission to the zygote, is translated and regulates development of the suspensor [46]. Similar events may also occur in *Plumbago zeylanica *[47].

### Limitations and future perspectives

Although this study focused on sequencing the small RNA population from isolated pollen grains, comprising just vegetative and sperm cells, it is clear that the number of reads (~58,000) was insufficiently deep, since many of the microRNAs were represented by just one or two reads, including the novel F-box targeting microRNA that was identified and validated in this study. Q-RT-PCR analysis of a range of pollen microRNAs has also confirmed that the depth of sequencing was not fully representative of the pollen small RNA transcriptome. One reason why this depth of sequencing was not saturating - even for just these two cell types - may be because of the major transcriptomic fluxes which clearly take place during male gametophyte development - affecting both the nucleus and organelles. Smaller RNAs from the degradation of transcripts generated by these events appear to constitute a high proportion of the transcriptome and may dilute out genuine small RNAs. Future work on pollen small RNAs will require a significantly higher number of reads.

The study is also limited as only the mature pollen stage of male gametophyte development was sequenced. Our results represent just a snapshot of small RNAs at a time when cell divisions have ceased and development is at a standstill, as the pollen becomes metabolically inactive and dehydrated prior to dispersal. The representation of several miR families only by shorter reads than the mature microRNA points to regulated degradation taking place [48]. To obtain a clear and comprehensive picture of the temporal dynamics of small RNA diversity, sequencing small RNAs from different isolated stages of male gametophyte development (unicellular microspore, bicellular pollen and early tricellular pollen) will be necessary. This will permit changes in coding transcript levels, as described in [28] and [29], to be correlated with changes in small RNAs. Analysis of small RNAs after pollen hydration and pollen tube growth would also be highly desirable.

## Conclusions

We have shown that even mature pollen - where male gametophyte development is temporarily arrested and cells are dehydrated prior to dispersal and the commencement of a new developmental programme (pollen tube growth) - shows a remarkable diversity of microRNAs and tasiRNAs. These small RNAs are likely to have contributed to the major transcriptomic changes earlier in development and may also be present to direct changes in transcript abundance and translation during pollen hydration, germination and tube growth - and even at syngamy and in immediate post-fertilisation development. The discovery of apparently pollen-specific microRNAs fulfils the expectation that this stage of development not only requires known somatically-expressed microRNAs but also uses stage-specific small RNAs.

## Methods

### Plant material

*Arabidopsis thaliana *ecotype Columbia-0 plants were grown in a controlled environment room (24 h light, 120-140 μmol/m2/sec, with 40-50% relative humidity, at 18-23°C). Mature pollen from flowering plants of the Columbia ecotype was collected as described in [17]. Plants of *Nicotiana tabacum *'Petit Havana' were grown to flowering and mature pollen was collected from freshly dehiscing anthers.

### RNA extractions

Total RNA from Arabidopsis mature pollen was extracted using the TRI Reagent^® ^method described in [17]. Total RNAs from 4-week-old seedlings and whole mature flowering plants of Columbia ecotype were extracted by the same method. For independent Q-RT-PCR verification of microRNA abundance, the small RNA fraction from Arabidopsis mature pollen was extracted using the miRVana kit (Ambion). To ensure that the total RNA sample was effectively free of contaminating sporophytic RNAs before its submission for 454 sequencing, a quality control PCR amplification of derived cDNA was performed to detect sporophyte-specific transcripts. Total RNA from *Nicotiana tabacum *pollen was extracted using the TRI Reagent^® ^method. The small RNA fraction was isolated using YM-100 size-exclusion filters (Millipore).

### 454 sequencing of small RNAs

The Arabidopsis mature pollen small RNA library was built and sequenced using 454 technology by Eurofins MWG (Ebersberg, Germany) using the following procedure:

#### Gel purification of small RNAs

Total RNAs were separated on a denaturating 15% polyacrylamide gel and stained with SYBRgreenII. As a molecular mass standard, a mixture of oligonucleotides with a size range of 19 and 29 nt was used. The small RNA fraction with a length of 19-29 bases was obtained by elution of the RNAs from the excised gel. The eluted RNAs were then precipitated and dissolved in water.

### cDNA synthesis

The gel-purified small RNAs were first poly(A)-tailed using a poly(A) polymerase followed by ligation of a RNA adapter to the 5' phosphate of small RNAs. First strand cDNA synthesis was then performed using an oligo(dT)-adapter primer and M-MLV-RNase H reverse transcriptase. Incubation temperatures were 42°C for 15 min, followed by 55°C for 5 min. The resulting cDNA was then PCR-amplified (20 cycles) to about 20-30 ng/μL using a high fidelity DNA polymerase.

#### Small RNA library sequencing

The PCR-amplified cDNAs corresponding to the small RNAs were then sequenced using the 454 Life Sciences (Roche) technology. More than 58000 reads were finally obtained.

### Bioinformatic analysis

#### Global BLAST analysis

31239 unique sequences derived from 54764 clipped sequences (with 5' and 3' adaptors removed) whose length was between 15 and 30 nt were used for a series of BLAST similarity searches (BLASTN). The BLAST output was analysed using a set of customized Perl/BioPerl scripts. A first set of BLAST searches against the Arabidopsis Chromosome sequences downloaded from the NCBI ftp://ftp.ncbi.nih.gov./genomes/Arabidopsis_thaliana was used to align all unique sequences to the Arabidopsis genome and to estimate the global level of identity (See Table 1). Then only sequences showing 0 or 1 mismatch with the Arabidopsis genome were considered for further analyses. These nucleotide sequences were then subjected to BLAST searches against the Arabidopsis intergenic (TAIR8_intergenic_20080228) and "genic" (TAIR8_seq_20080412) nucleotide sequence databases downloaded from TAIR http://www.arabidopsis.org/. For each individual sequence the best hits and corresponding scores for each genomic region were extracted from the BLAST alignments. To assign any sequence to a single chromosomal location (intergenic hit) or a known transcript ("genic" hit), the best scores for genic and intergenic BLAST searches were compared and assigned based on the higher score. Sequences having identical scores for the two types of genomic locations were classified into an "undetermined" category (See Table 3). After that, sequences having a "genic" hit were sorted into different categories: silencing RNAs (known miRNAs and TAS genes), candidate silencing RNAs (protein coding genes, transposons and pseudogene), non-protein-coding genes (mainly rRNAs and tRNAs plus snoRNAs and snRNAs) and organellar small RNAs, corresponding to chloroplast and mitochondrial sequences.

#### Known microRNA identification

To enhance the identification of known microRNAs, a specific program was developed to directly compare all the individual nucleotide sequences obtained by 454 sequencing against the Arabidopsis microRNA database (miRNA_family.fasta) downloaded from the Arabidopsis Small RNA Project website http://asrp.cgrb.oregonstate.edu/db/. Less stringent parameters were then selected such that incomplete sequences (but perfectly matching the miRNA sequence), as well as sequences showing up to 2 mismatches were considered (see Table 4).

#### Novel microRNA identification

To detect new microRNAs in our Arabidopsis Col-0 mature pollen sample, we used MiRCat [49]. All the 31239 unique sequences were first submitted to the MiRCat software using default parameters, which first BLASTs each individual sequence against the Arabidopsis thaliana genome (TAIR v 7.0) requiring a 100%. Then the software evaluates the possibility that the extended sequence (upstream and downstream) can form a hairpin structure. Good candidate sequences are then submitted to a target prediction tool, which attempts to find putative target(s) for the small RNA candidate by measuring small RNA/mRNA sequence complementarity. Only small RNA sequences that could form a predicted hairpin structure and having a putative mRNA target were considered for further molecular analysis. Predicted mRNA targets for modified 5' RACE analysis to detect cleaved products resulting from predicted miRNA action (see below) were selected on the basis of their expression patterns in the male gametophyte [50].

### TasiRNA analysis

To detect phased small RNA clusters corresponding to tasiRNAs, the pssRNAMiner software http://bioinfo3.noble.org/pssRNAMiner was used [51]. Only small RNAs matching the transcript coding part of the nuclear genome (excluding all non-protein coding structural RNAs and organellar small RNAs) were used as input and matched against the *Arabidopsis *genome. Using a maximum offset position of 1 and a mapping ambiguity of 6, the software then identified phased small RNA clusters by evaluating P-values of hypergeometric distribution. A minimum p-value of 0.05 was used to detect statistically significant clusters.

To determine whether *TAS *precursors were expressed in mature pollen, RT-PCRs were performed with cDNA synthesized from 2 ng total RNA using a Retroscript kit (Ambion) with random primers, from mature pollen and a mature plant control. Specific primers used to detect TAS transcripts are described in the Additional file 1: Table S3.

### 5' RACE to detect cleaved mRNAs

Isolation of polyA RNA from total RNA of *A. thaliana *Col-0 material and generation of 5' RACE cDNA was performed as described in [17]. To detect the predicted cleaved products of mRNAs that are putatively targeted by the candidates for novel microRNAs identified in the bioinformatic analysis, 1 μl cDNA was amplified using a gene-specific primary primer (designed according to GeneRacer kit criteria) and the 5' RACE GeneRacer primer according to GeneRacer kit instructions. 1 μl of the product from this PCR reaction was subsequently used in a nested PCR amplification using a gene-specific nested primer (designed according to GeneRacer kit criteria) and the 5' RACE GeneRacer nested primer according to GeneRacer kit instructions. Reactions were set up with two controls, one control with all components but lacking the gene-specific primer and another control lacking the GeneRacer primer. Primers sequences used in this study are available in the Additional file 1: Table S3. Products of the nested reaction were run out on 3% agarose gels and if products corresponding to the approximate size range were detected the gel section was excised and PCR fragments extracted using the Qiaex II kit (Qiagen). PCR fragments were cloned into sequencing vectors (pJet & pGemT-Easy) and sequenced. Cleavage points of the amplified transcript were identified using VecScreen http://www.ncbi.nlm.nih.gov/VecScreen/. The same modified 5' RACE analysis was extended to cover known miRNA binding sites on the same transcripts, as several of the predicted mRNA targets are validated targets of previously described miRNAs.

### Quantitative RT-PCRs of microRNAs

We performed quantitative RT-PCR to validate the relative abundance of microRNAs identified with 454 sequencing in 2 independent samples of Col-0 mature pollen and leaf. Small RNAs from these samples were extracted and isolated using the miRVana kit (Ambion) and corresponding cDNAs were synthesized using the QuantiMiR kit (SBI). Quantitative PCR was performed on a MJ Research Chromo4 machine, using SYBRGreen JumpStart Taq ReadyMix (Sigma-Aldrich). Primers were defined for a subset of 16 known miRNAs and a putatively new miRNA (see Additional file 1: Table S3). A standard curve was performed for each miRNA using a dilution series of cDNA products (1/5, 1/25, 1/125, and 1/625) to estimate PCR efficiency [52]. All PCR reactions were performed in duplicate. A fixed dilution of cDNA was used for both leaf and mature pollen samples to generate a Ct value. The relative abundance of each miRNA within mature pollen sample was estimated by comparing the Ct value difference between the highly expressed miRNA (miR156). These values were then converted into a fold-change difference (1 Ct = 2-fold change). To estimate the relative abundance of miRNAs in sporophytic and gametophytic samples, the Ct value was directly compared and transformed into a fold-change difference.

### Expression of conserved small RNAs in bicellular tobacco pollen

Small RNA cDNA synthesis and end-point RT-PCRs of miRNAs were performed as described in [17] for 78 ng of the small RNA fraction from mature *Nicotiana tabacum *pollen, using developing floral bud material as control. We amplified *N. tabacum *homologues of *Arabidopsis *microRNAs that were found in this study using specific primers (see Additional file 1: Table S3), to determine conservation of pollen expression. Primers for amplification of *N. tabacum *small RNAs were designed from described *N. tabacum *small RNAs or from highly conserved dicotyledonous sequences from MiRBase http://www.mirbase.org/.

## Authors' contributions

Experimental strategy was jointly devised by DT, GLeT, HGD and RG-D. GLeT and RG-D carried out the bulk of the experimental work and data analysis, assisted in the latter by RS. JER, SH and SM carried out some of the experiments and provided materials and reagents. GLeT and RG-D jointly wrote the manuscript which has been read and approved by all authors.

## Supplementary Material

Additional file 1 **Additional Tables and figures**. Table S1: Examples of sequencing mismatches resulting from 454 analysis. Table S2: Microarray expression values of miRNA targets; SUVH6, SUVH5 and F-Box protein family. Table S3: Primers used in this study. Figure S1: MicroRNA amplification by quantitative RT-PCR. Figure S2: SUVH6 transcript cleavage points detected using 5'RACE-PCR. Figure S3: Trans-acting siRNA features of TAS1A, TAS1B, TAS1C and TAS2 transcripts. Figure S4: Expression of *TAS *precursors in mature plants and mature pollen. Figure S5: Expression of small RNAs in mature bicellular pollen of tobacco. Click here for file
