# Supplementary material for: MicroRNA and tasiRNA diversity in mature pollen of Arabidopsis thaliana
Source: BMC Genomics. 2009 Dec 30;10:643. doi: 10.1186/1471-2164-10-643 (PMC2808329; doi:10.1186/1471-2164-10-643)
Supplement: Additional file 1 — Additional Tables and figures. Table S1: Examples of sequencing mismatches resulting from 454 analysis. Table S2: Microarray expression values of miRNA targets; SUVH6, SUVH5 and F-Box protein family. Table S3: Primers used in this study. Figure S1: MicroRNA amplification by quantitative RT-PCR. Figure S2: SUVH6 transcript cleavage points detected using 5'RACE-PCR. Figure S3: Trans-acting siRNA features of TAS1A, TAS1B, TAS1C and TAS2 transcripts. Figure S4: Expression of TAS precursors in mature plants and mature pollen. Figure S5: Expression of small RNAs in mature bicellular pollen of tobacco. [file 1471-2164-10-643-S1.DOC]

**Additional file 1**

**Supplemental tables and Figures**

**Supplemental Tables S1- S3**

**Supplemental Figures S1-S5**

**Additional Tables**

**Table S1: Examples of sequencing mismatches resulting from 454 analysis.**

Format: Word 2003 doc.

**Table S2: Microarray expression values of miRNA targets; SUVH6, SUVH5 and F-Box protein family.**

Format: Word 2003 doc.

**Table S3: Primers used in this study.**

Format: Word 2003 doc.

**Additional Figures**

**Figure S1: MicroRNA amplification by quantitative RT-PCR.**

Format: Word 2003 doc.

**Figure S2: SUVH6 transcripts cleavage points detected using 5’RACE-PCR.**

Format: Word 2003 doc.

**Figure S3: Trans-acting siRNA features of TAS1A, TAS1B, TAS1C and TAS2** **transcripts.**

Format: Word 2003 doc.

**Figure S4: Expression of *TAS* precursors in mature plants and mature pollen.**

Format: Word 2003 doc.

**Figure S5: Expression of small RNAs in mature bicellular pollen of tobacco.**

Format: Word 2003 doc.

**Additional Tables**

**Table S1 – Examples of sequencing mismatches observed with 454.** Comparison of miR162, miR165. miR173 and miR773 sequences analyzed by Q-RT-PCR (‘Correct sequence’) and 454 sequencing

|  | **Correct sequence** | **454 sequence** | **Difference** |
| --- | --- | --- | --- |
| **miR162** | TCGATAAACCTCTGCATCCAG | TCGATAAACCTCTGC**_**TCCA**X** | 1 deletion, 1 missing |
| **miR165** | TCGGACCAGGCTTCATCCCCC | TCGGACCAGGCTTCA**A**T**A**CCCC**X** | 2 insertions, 1 missing |
| **miR173** | TTCGCTTGCAGAGAGAAATCAC | TTCGCTTGCAGAGA**A**GAAA**A**TCAC | 2 insertions |
|  |  | TTCGCTTGCAGAGAGAAA**A**TCA**X** | 1 insertion, 1 missing |
| **miR773** | TTTGCTTCCAGCTTTTGTCTC | TTTG**A**CTTCCAGCTTTT**TA**GT**XXX** | 3 insertions, 3 missing |

**Table S2 – Microarray expression values of miRNA targets; SUVH6, SUVH5 and F-Box protein family.** Pollen developmental data [29] were normalized using MAS5.0 software (P: Present, M: Marginal and A: Absent). Sperm cell data [18] were normalized using GCOS1.4 software (P: Present, A: Absent).

**Pollen developmental data** (29)

|  |  | **UNM** | **BCP** | **TCP** | **MP** |
| --- | --- | --- | --- | --- | --- |
| **At3g19890** | F-Box protein family | 272.4 (PP) | 250.2 (PA) | 69.57 (MA) | 57.95 (AA) |
| **At2g22740** | SUVH6 | 471.1 (PP) | 499.8 (PP) | 849.2 (PP) | 866 (PP) |
| **At2g35160** | SUVH5 | 310.1 (PP) | 276.1 (PP) | 250.4 (AP) | 256.7 (AP) |

**Sperm cell data** (18)

|  |  | **Sperm Cell** | **Mature pollen** | **Seedling** |
| --- | --- | --- | --- | --- |
| **At3g19890** | F-Box protein family | 255.9 (P) | 28.9 (A) | 5.0 (A) |
| **At2g22740** | SUVH6 | 213.5 (P) | 368.0 (P) | 266.5 (P) |
| **At2g35160** | SUVH5 | 2436.8 (P) | 114.5 (P) | 71.3 (A) |

**Table S3 – Primers used in this study.**

| **Primers for *TAS* genes** | **Sequence 5’→3’** |
| --- | --- |
| *TAS1A* FORWARD | GCAAGTACAATCTCATCTTAACTCAA |
| *TAS1B* FORWARD | TAACTCCATCTTAACACAAAAGTTGA |
| *TAS1C* FORWARD | AGCGTCGTCTATAGTTAGTTTGAGAT |
| *TAS1* REVERSE | TACGCTATGTTGGACTTAGAATA |
| *TAS2* FORWARD | TGAGTTTACGAGTTACAAGTTGGTT |
| *TAS2* REVERSE | ATCGTGTTTCAAAATACACAAGTTC |

| **Gene-specific reverse primers**  **For 5’ RACE** | **Sequence 5’→3’** |
| --- | --- |
| At3g19890 primary | CACAAGTGCGCTCCGATACACGTCTTTT |
| At3g19890 nested  (to detect ath-MIR2939 cleavage) | GGTTCTTGTCCTTGTCGTAACCGAGAGCAA |
| At3g19890 nested  (to detect miR774 cleavage) | CGGACCTCCACAGCTTCTCCAAGAACCTCTTT |
| At3g17265 primary | GTCCACCAAGAAACTCAACGGCTCACTTG |
| At3g17265 nested  (to detect ath-MIR2939 cleavage) | CCTTCAAATTCACGCCATTTGAGCGTATGA |
| At1g27880 primary | GCGACCAGCACGTCCAATTTCCTGAAC |
| At1g27880 nested | CGCAGGAAGACCACTGTGATAACCCTTTGC |
| At3g28140 primary | CAGCCACAGAACAAAACGGAACCCAAGA |
| At3g28140 nested | AAAGAGACGGCGTCGGCGAAAGAAAC |
| At3g12890 primary | TTCGTGAAGCCATCCGTCATCGTGTTC |
| At3g12890 nested | CCTCCACCGAATAGCGTCCCACTTTTG |
| At1g30330 primary  (to detect miR167 cleavage) | TGAACCCCCAACTGATGCGATACCGTTAC |
| At1g30330 nested  (to detect miR167 cleavage) | CGGTTCCCAAAGTCGTCACAGTTCCCGTTG |
| At1g30330 primary  (to detect Ath-miRLC1 cleavage) | AAATGGAGGCAACGAGACAGCGTTTGG |
| At1g30330 nested  (to detect Ath-miRLC1 cleavage) | AGTGGTGACGTGTTGGGCTGAGAAGCA |
| At2g35160 primary  (to detect miR778 cleavage) | GGATGGACCTCCACCATATTTTGCTCACTTG |
| At2g35160 nested  (to detect miR778 cleavage) | GCATCACGTTTCCGCCTTGACCAGTGT |
| At2g35160 primary  (to detect ath-MIR2936 cleavage) | TGCTTTTCCTGCAAACAGACCCCATCA |
| At2g35160 nested  (to detect ath-MIR2936 cleavage) | GGATGGACCTCCACCATATTTTGCTCACTTG |
| At2g22740 primary | ACAAGCGCACACCCTCGCTTCAGCTT |
| At2g22740 nested 1  (to detect miR778 cleavage) | GCATCACGTTTCCGCCTTGACCAGTGT |
| At2g22740 nested 2  (to detect ath-MIR2934 cleavage) | CATCACCAACCTCAACCCCAGGCACTT |
| At3g63350 primary | CGTCAAGCTCCCTCTCCACACCATCAG |
| At3g63350 nested | CATGGCTTGGACGTAGCCTCTCGCTCTT |
| At2g40470 primary | AATCCGGCAACTTGGGAGTTGGAAGGT |
| At2g40470 nested | CCGTGAGCTCGGCTTGTAAAGCTTGGA |

| **Forward primers for *Arabidopsis* miRNAs amplification** | **Sequence 5’→ 3’** |
| --- | --- |
| miR156 | TGACAGAAGAGAGTGAGCAC |
| miR161 | TTGAAAGTGACTACATCGGGG |
| miR159 | TTTGGATTGAAGGGAGCTCTA |
| miR158 | TCCCAAATGTAGACAAAGCA |
| miR173 | TTCGCTTGCAGAGAGAAATCAC |
| miR403 | TTAGATTCACGCACAAACTCG |
| miR157 | TGGCTTGGTTTATGTACACCG |
| miR160 | TGCCTGGCTCCCTGTATGCCA |
| miR824 | TAGACCATTTGTGAGAAGGGA |
| miR165 | TCGGACCAGGCTTCATCCCCC |
| miR845 | TCGCTCTGATACCAAATTGATG |
| miR162 | TCGATAAACCTCTGCATCCAG |
| miR171a | TGATTGAGCCGCGCCAATATC |
| miR171b,c | TTGAGCCGTGCCAATATCACG |
| miR773 | TTTGCTTCCAGCTTTTGTCTC |
| ath-MIR2939 | TAACGCACAACACTAAGCCAT |
| miR774 | TTGGTTACCCATATGGCCATC |

| **Forward primers for tobacco small RNA amplification** | **Sequence 5’→ 3’** |
| --- | --- |
| miR159 | TTTGGATTGAAGGGAGCTCTA |
| miR160 | TGCCTGGCTCCCTGTATGCCA |
| miR162 | TCGATAAACCTCTGCATCCAG |
| miR164 | TGGAGAAGCAGGGCACGTGCA |
| miR166 | TTTGAGGGGAATGTTGTCTGG |
| miR167 | TGAAGCTGCCAGCATGATCTGG |
| miR168 | TCGCTTGGTGCAGGTCGGGAA |
| miR172 | AGAATCTTGATGATGCTGCAT |
| miR396 | TTCCACAGCTTTCTTGAACT |
| miR399 | tgccaaaggagagttgcccta |
| NRS11 | TGCCAAAGGAGAGTTGCCCTA |

**Additional Figures**

**Figure S1 – microRNAs amplification by quantitative RT-PCR.**

Q-RT-PCR products of 16 known microRNAs and a putative novel microRNA

(ath-MIR2939) in mature pollen (MP) and leaf (L) samples.

**
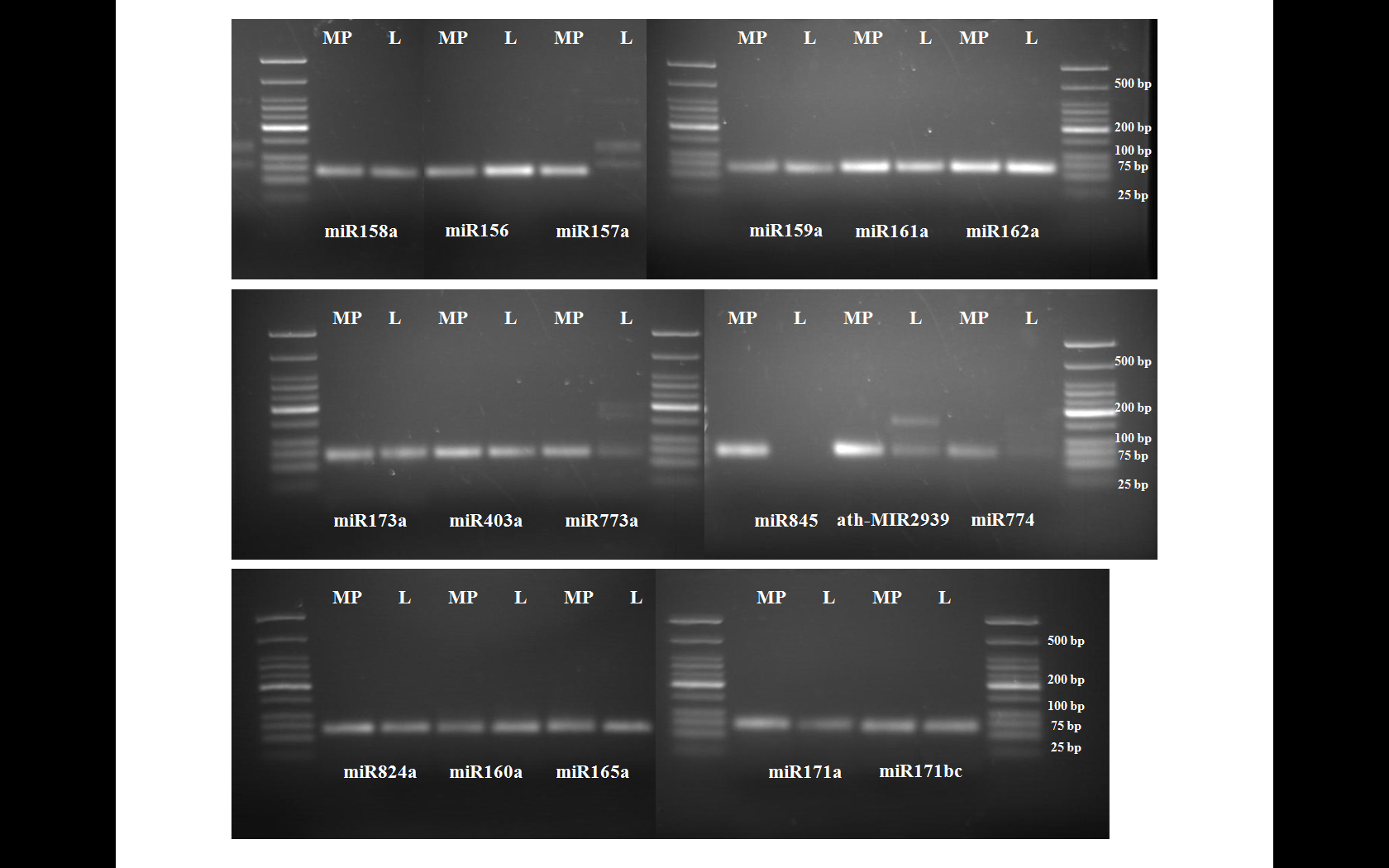
**

**Figure S2 – SUVH6 transcripts cleavage points detected using 5’RACE-PCR.**


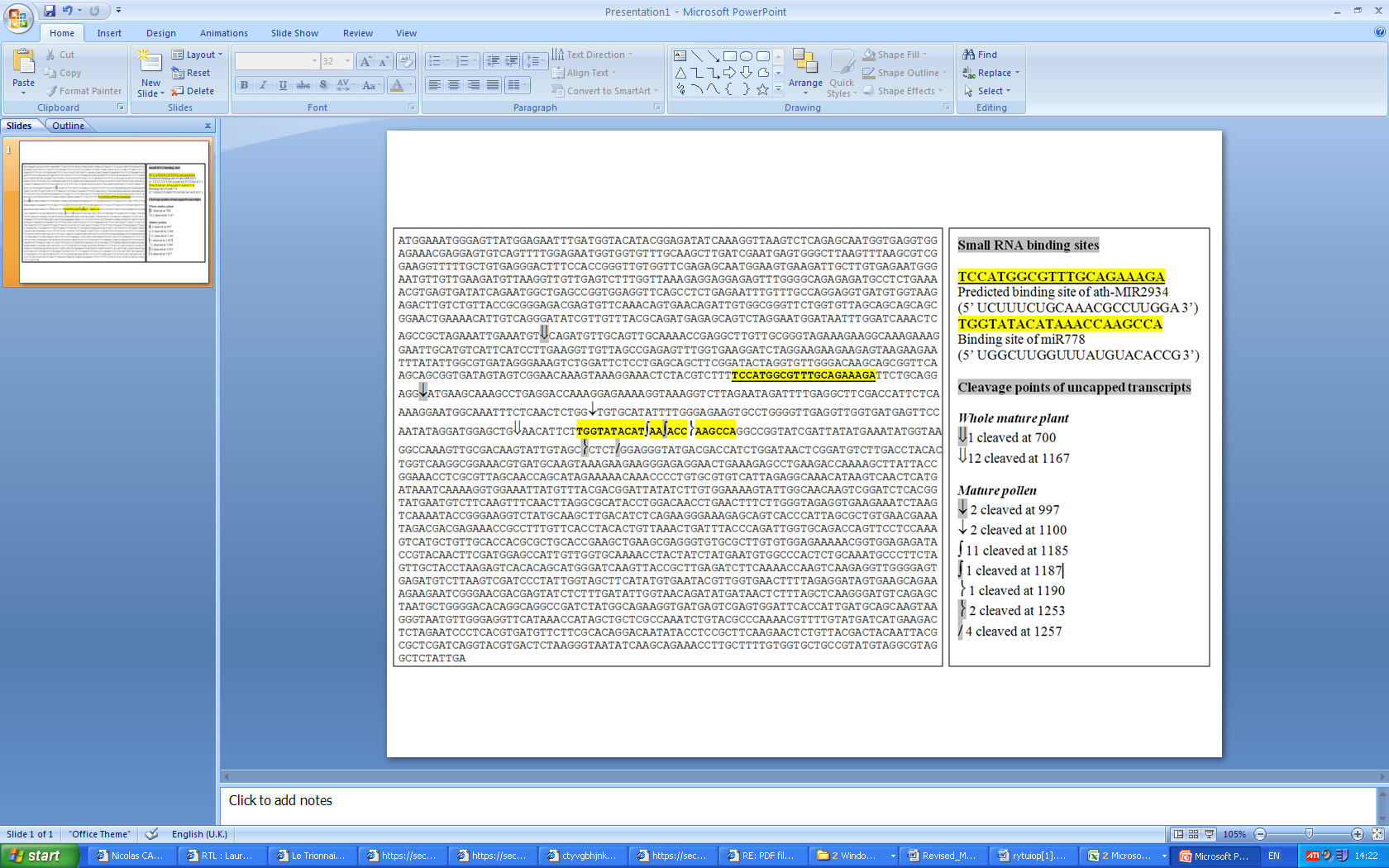
**Small RNA binding sites** on the *SUVH6* full length coding sequence for both **miR778** and the putative new miRNA ath-MIR2934 are highlighted in yellow. The corresponding **points of cleavage** of uncapped transcripts as detected by modified 5’ RACE in mature pollen and whole mature plant material are indicated by different types of arrow.

**Figure S3 – Trans-acting siRNA features of TAS1A, TAS1B, TAS1C and TAS2 transcripts**

**
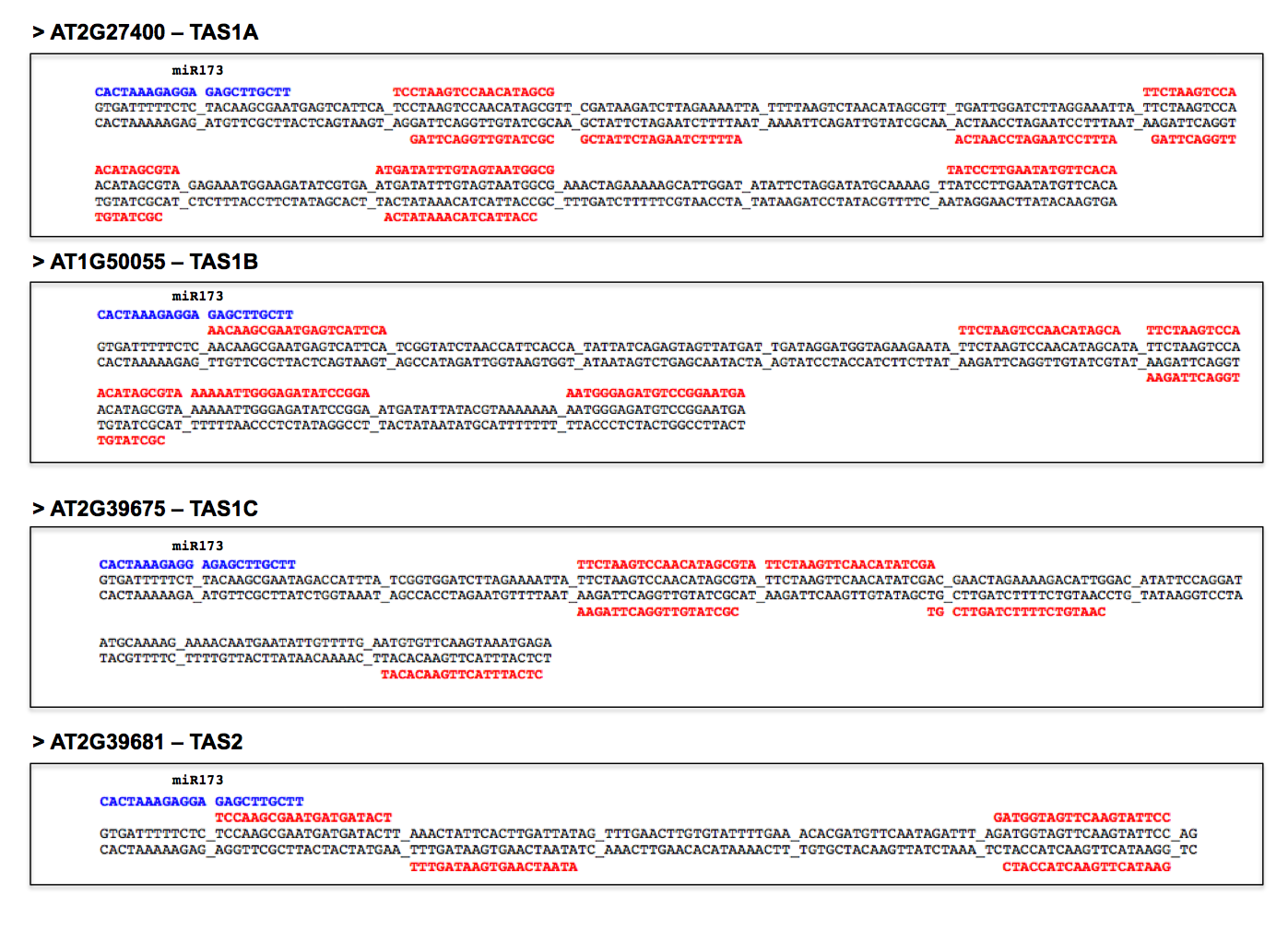
**

Small phased RNAs perfectly matching *TAS1A, B, C* and *TAS2* are indicated in **red**. miR173, which is known to be the phase-initiator for trans-acting siRNA is shown in **blue** in TAS transcripts.

**Figure S4 – Expression of *TAS* precursors in mature plant and mature pollen**

**
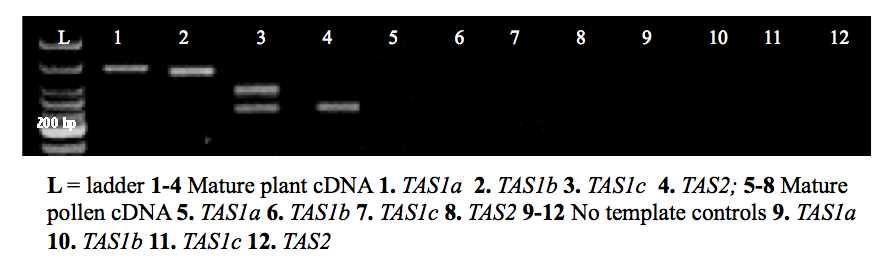
**

*TAS1A, B, C* and *TAS2* amplified from Col-0 mature plant material (1, 2, 3 and 4). These precursors are undetectable in mature pollen (5, 6, 7 and 8).

**Figure S5 – Expression of small RNAs in mature bicellular pollen of tobacco**

**
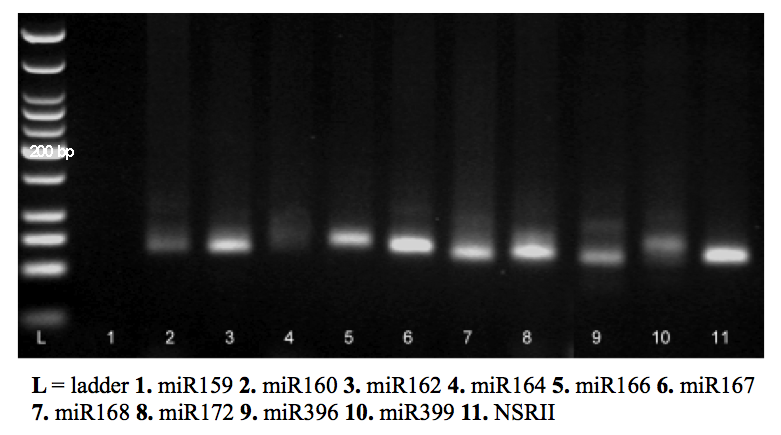
**

RT-PCR to reveal the presence of 11 microRNAs in tobacco mature pollen using small RNA samples. Only miR159 cannot be detected.
